# Hospital‐Associated Antimicrobial Resistant Bacteria on 95 Mobile Phones: An International Metagenomic “Phonome” Analysis

**DOI:** 10.1002/mbo3.70321

**Published:** 2026-06-18

**Authors:** Adrian Goldsworthy, Matthew Olsen, Nchafatso G. Obonyo, Peter Jones, Simon McKirdy, Abiola Senok, Rashed Alghafri, Rose Ghemrawi, Reem Almheiri, Oystein Tronstad, Jacky Y. Suen, John F. Fraser, Lotti Tajouri

**Affiliations:** ^1^ Critical Care Research Group, The Prince Charles Hospital Brisbane Queensland Australia; ^2^ Wesley Research Group Brisbane Queensland Australia; ^3^ Institute for Molecular Bioscience The University of Queensland Brisbane Queensland Australia; ^4^ Harry Butler Institute Murdoch University Murdoch Queensland Australia; ^5^ Faculty of Health Sciences and Medicine Bond University Gold Coast Queensland Australia; ^6^ KEMRI‐Wellcome Trust Research Programme and Initiative to Develop African Research Leaders Kilifi Kenya; ^7^ Wellcome Trust Centre for Global Health Research, Imperial College London London UK; ^8^ College of Medicine Mohammed Bin Rashid University of Medicine and Health Sciences Dubai UAE; ^9^ International Centre for Forensics, Dubai Police Dubai UAE; ^10^ AAU Health and Biomedical Research Center Al Ain University Abu Dhabi UAE; ^11^ Physiotherapy Department The Prince Charles Hospital Brisbane Queensland Australia; ^12^ Dubai Police Scientist Council, Dubai Police Dubai UAE

**Keywords:** antimicrobial resistance, bacteria, fomite, infection, metagenomics

## Abstract

Antimicrobial resistant healthcare‐associated infections present an increasing threat to public safety and the sustainability of healthcare systems around the world. Mobile phones have been highlighted as a fomite that negates hand hygiene and contributes to the dissemination of pathogenic microorganisms in healthcare settings. The objective of the current stidy was to investigate the presence of bacteria, antimicrobial resistance and virulence genes associated with high morbidity on 95 mobile phones within healthcare settings. Next‐Generation Metagenomic Sequencing was undertaken and FastQ files were subsequently analyzed within COSMOSid to enable taxonomic identification. Antibiotic resistant genes, virulence genes and bacteriophages were co‐located with bacteria associated with the highest global mortality. Antibiotic resistant genes were manually annotated and cross referenced with the Comprehensive Antibiotic Resistance Database (CARD) to identify gene–drug interactions. On average, mobile phones were identified to be contaminated with 3.62 of the top 10 highest mortality‐causing bacteria and 2.49 ESKAPE pathogens. A total of 262 unique ARGs, 448 unique VFGs, and 314 bacteriophages were identified. Mobile phones within healthcare settings harbor pathogens alongside genes associated with increased virulence and antimicrobial resistance. Additionally, mobile phones, known to be infrequently sanitized, may increase antimicrobial resistance by providing a contaminated platform which facilitates continued horizontal genetic transfer.

## Graphical abstract

- On average, phones harbored 3.62 of the top 10 killers and 2.49 ESKAPE organisms.
- In total, 667 VFGs (8621 Hits) and 262 ARGs (2475 Hits) were identified on 95 mobile phones.
- Mobile phones serve as a platform for horizontal gene transfer and AMR development.

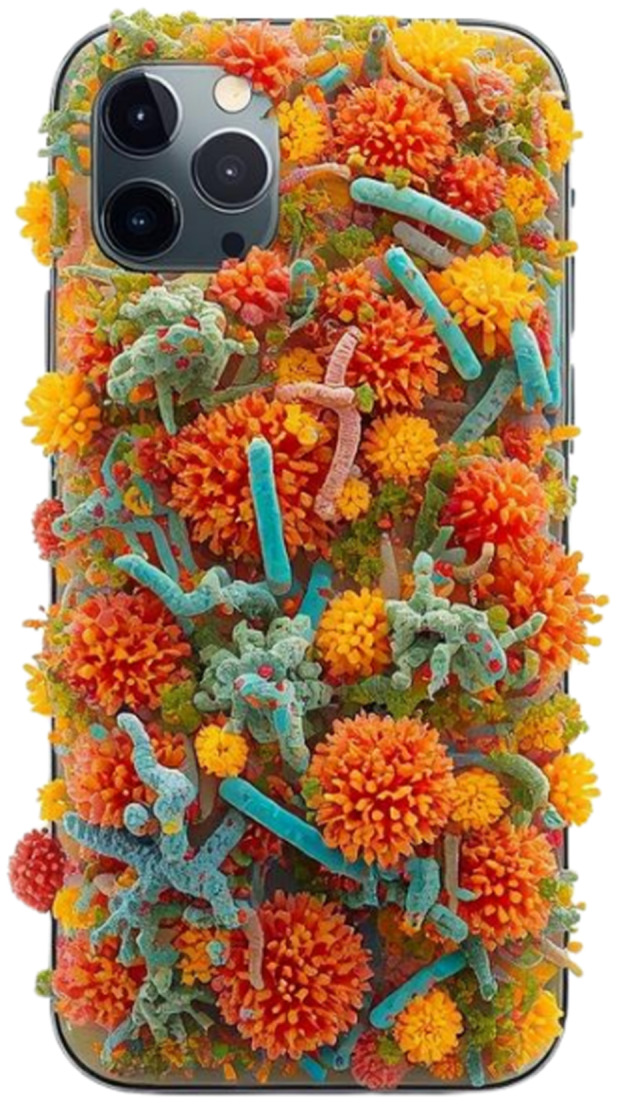

## 1 Introduction

In 2019, an estimated 13.7 million deaths were attributable to bacterial infections, contributing to approximately 704 million disability‐adjusted life‐years globally (Ikuta et al. 2022; Naghavi et al. 2024). The majority of these fatalities have been linked to 10 bacterial species inclusive of 5 gram‐negative bacteria (*Escherichia coli, Klebsiella pneumoniae, Pseudomonas aeruginosa, Acinetobacter baumannii, and Enterobacter* spp.) and 5 gram‐positive bacteria (*Staphylococcus aureus*, *Streptococcus pneumoniae*, *Group B Streptococcus*, *Enterococcus faecalis*, and *Enterococcus faecium*). These bacteria, inclusive of all ESKAPE pathogens, also encompass nine bacterial species designated by the World Health Organization (WHO) as “2024 Global Priority Pathogens” (World Health Organization 2024). The morbidity and mortality associated with these pathogens relate to their high virulence, resistance to antimicrobial drugs, and low treatability. Characteristics which are associated with the presence of antimicrobial resistance genes (ARGs) and virulence factor genes (VFGs) in microbial genomes.

Mobile phones have become integral to contemporary clinical practice, serving multiple functions including communication, data access, clinical assessment (e.g., illumination and photography), and integration with digital tools such as virtual reality headsets and ultrasound devices (Goldsworthy et al. 2024; García et al. 2025; Júnior 2022). However, their high potential for contamination resulting from widespread use, including frequent use within toilets, and infrequent disinfection in clinical environments, raises concerns regarding their potential role as vectors for microbial contamination and healthcare‐acquired infections (HAIs) (Olsen et al. 2021). In a recent metagenomic study at the 2023 World Organization for Family Doctors conference in Sydney (Australia), 882 bacteria, 1229 viruses, and 88 fungi, in addition to 65 unique antibiotic‐resistant genes and 86 VFGs, were identified on 20 attendees' mobile phones highlighting their potential role as both reservoirs for HAIs and a hazardous fomite warranting attention in healthcare settings (Olsen et al. 2025). Bacteria known to be associated with HAIs and viruses such as SARS‐CoV‐2 have been demonstrated to persist on mobile phones for at least 28 days. This provides not only adequate time‐related opportunity for horizontal genetic transfer, accelerating the development of pathogenic and antimicrobial resistant microbes, but also favors their transmission and dissemination throughout the hospital environment (Olsen, Demaneuf, et al. 2023; Simmonds‐Cavanagh 2022).

The increased potential for patients to contract HAIs places increased pressure on increasingly under‐resourced healthcare systems (Gidey et al. 2023). In Europe alone, HAIs account for over 25 million hospital bed days per year at a cost of between 13 and 24 billion Euros annually (Pittet 2025). Clinically, increases in antimicrobial resistance have impacted patient management, with many first‐line antibiotics rendered ineffective and a rising number of deaths attributable to infections caused by untreatable, antibiotic‐resistant bacteria (Ventola 2015).

Next‐generation metagenomic sequencing has emerged as a powerful tool for identifying ARGs and VFGs within complex microbial communities (Osman et al. 2024; Martiny et al. 2024). Unlike culture‐based methods, metagenomic sequencing enables the simultaneous detection of a broad range of microbial DNA, allowing for high‐resolution characterization of both the taxonomic composition and functional potential of microbial populations (Osman et al. 2024). Increasingly, co‐occurrence analyses, where ARGs and VFGs are found in specific chromosomal/extrachromosomal bacterial genomes or mobile genetic elements (MGEs), are being used to identify microbial genes with direct clinical relevance (Martiny et al. 2024; Mustapha et al. 2022). This approach not only provides insight into the resistome and virulome associated with specific pathogens within an environment but also provides insight into the potential for horizontal genetic transfer, amplifying the potential for the rapid emergence and dissemination of antibiotic resistant organisms (Brito 2021).

Although a growing number of metagenomic investigations have identified high levels of microbial contamination on healthcare workers' mobile phones, there remains a lack of comprehensive synthesis evaluating whether these devices serve as reservoirs for high‐mortality HAI‐associated pathogens. To date, individual metagenomic studies of healthcare‐associated mobile phones have utilized relatively small sample sizes. This study aims to address these gaps by conducting a robust analysis of metagenomic data derived from swabs of healthcare workers' mobile phones. Specifically, the analysis will focus on the prevalence of the top 10 bacterial species associated with the highest global mortality and their co‐localization with antimicrobial resistant genes (ARGs), VFGs, and bacteriophages indicative of enhanced pathogenic potential and reduced therapeutic options.

## 2 Methods

### 2.1 Sample Collection and Population

Metagenomic data from studies investigating microbial contamination of 95 mobile phones of hospital healthcare workers from Australia (*n* = 56) and the United Arab Emirates (UAE) (*n* = 39) were analyzed (Tajouri et al. 2021; Boucherabine, Nasar, Mohamed, et al. 2022; Boucherabine, Nasar, Zaher, et al. 2022; Olsen et al. 2022) (SRA numbers available in Table S1). Studies were primarily from either pediatric general wards or emergency departments, with a small number of samples relating to the neonatal intensive care unit (NICU; *n* = 5) and pediatric intensive care unit (PICU; *n* = 5) in an Australian hospital. Despite the studies' small sample size, samples from the NICU and PICU displayed higher levels of pathogenic organisms than the general pediatric ward (Tajouri et al. 2021). The majority of samples (39 from UAE and 26 from Australia) were directly processed following swabbing of mobile phones for DNA extraction and downstream DNA next‐generation sequencing (“direct sequencing”). A total of 30 additional mobile phone derived swab samples from healthcare workers in Australia were subject to culture on agar plates with colonies pooled prior to DNA extraction and subsequent DNA next‐generation sequencing (“indirect sequencing”). Complete details regarding sampling, processing, and sequencing methodology have been published elsewhere (Tajouri et al. 2021; Boucherabine, Nasar, Mohamed, et al. 2022; Boucherabine, Nasar, Zaher, et al. 2022; Olsen et al. 2022).

### 2.2 Sequencing and Analysis

Briefly, the raw sequenced data set Fastq files from all 95 samples were analyzed using the CosmosID software to identify bacteria, fungi, VFGs, and antibiotic resistance genes. The data sets were then analyzed with proper mining bio‐informatic analytic tools using high‐performance data‐mining k‐mer algorithms and highly dynamic comparator databases (GenBook). The overall database was derived from curated GenBook Databases comprising over 150,000 bacteria, viruses, fungi, and protists genomes and gene sequences from both private and public sources, including the National Center for Biotechnology Information, the Pathosystems Resource Integration Center, DNA Data Bank of Japan, M5nr, Integrated Microbial Genomes and Microbiomes, and the European Nucleotide Archive. The resultant data were filtered using a multikingdom resolutive taxonomic identification analysis within CosmosID. This filtering was based on internal statistical scores from CosmosID, which enabled the listing of results without further validation to determine their presence in each sample. Richness was reported as the number of distinct microbes or genes found in all mobile phones, while hits describe the repeated number of distinct microbes found across all 95 mobile phones.

Finally, data were collated in Microsoft Excel to enable the co‐location of target bacteria associated with the highest global mortality (*S. aureus*, *E. coli*, *S. pneumoniae*, *K. pneumoniae*, *P. aeruginosa*, *A. baumannii*, *Enterobacter* spp., *Group B Streptococcus*, *E. faecalis*, and *E. faecium*) with ARGs, VFGs, and bacteriophages on the same mobile phones. This co‐location, whilst not providing direct evidence, highlights the potential for increased antibiotic resistance and virulence. Antibiotic resistant genes co‐located with a specific target bacteria ≥ 10% of the time were manually annotated, cross‐referenced with the Comprehensive Antibiotic Resistance Database (CARD) to identify gene–drug interactions, and included in the final analysis (Alcock et al. 2023).

## 3 Results

### 3.1 Overview of Samples

Direct sequencing depth ranged from 33 to 156 Mbp (mean: 53 Mbp) for Australian samples and 7.9 to 116 Mbp (mean: 42.5 Mbp) for UAE samples, while the indirect Australian sequenced samples ranged from 4.5 to 17.6 Mbp (mean: 7.5 Mbp). Sequencing of the 95 mobile phone derived samples led to the identification of 1839 distinct bacterial strains (8917 Hits), 563 bacteriophages (6428 Hits), 667 VFGs (8621), and 262 ARGs (2475 Hits). Gram‐negative and gram‐positive bacterial richness accounted for 47.5% (872/1839) and 52.5% (966/1839), respectively, while their total respective hit numbers were similar at 48.4% (4309/8917) and 51.6% (4598/8917).

### 3.2 Prevalence of Contamination on Healthcare Worker Mobile Phones

All mobile phones were contaminated with both gram‐negative (total Hits: 243, average Hits: 2.56) and gram‐positive bacteria (total Hits: 120, average Hits: 1.26), with several bacteria identified as being associated with one or more of the top 10 highest mortality‐causing bacterial pathogens globally (Table 1) (Ikuta et al. 2022). Samples from Australia and the UAE exhibited different metagenomic profiles, both in the types of bacteria present on phones and in their co‐localization of resistome‐ and virulome‐associated genes. In particular, *P. aeruginosa* was identified in all UAE samples, consistently co‐located with genes encoding efflux pumps known to confer multidrug resistance, including reduced susceptibility to beta‐lactams, fluoroquinolones, and macrolides. Of note, 75% of the 95 mobile phones had more than one high‐mortality‐associated bacteria, with seven having all five gram‐negative bacteria. Three bacteria, *S. aureus*, *P. aeruginosa*, and *Group B Streptococcus*, were identified on over 50% of mobile phones (Table 1). Conversely, *K. pneumoniae*, *E. faecalis*, and *E. faecium* were identified on less than 15% of devices. On average, mobile phones were found to be contaminated with 3.62 of the top 10 highest mortality‐causing bacteria and 2.49 ESKAPE pathogens, highlighting the ability for mobile phones to act as a reservoir for microbes known to cause HAIs. Additionally, bacteria associated with the HACEK group including *Haemophilus* spp. (12 Hits), *Aggregatibacter* spp. (27 Hits), *Cardiobacterium hominis* (12 Hits), *Eikenella corrodens* (6 Hits), and *Kingella* spp. (13 Hits) were identified. Of note, all bacteria associated with the WHO medium, high, and critical priority pathogens list, including *Listeria monocytogenes* (21%), *Neisseria meningitidis* (8%), *Clostridioides difficile* (7%), and *Salmonella* (5%) were identified on mobile phone samples.

**Table 1:** Relative presence of antibiotic‐resistant genes.

Microorganism, number of phones (Relative percentage)	Antibiotics affected		Number of related mechanisms identified	Number of related genes (Hits)	Average number of genes per phone
*Pseudomonas aeruginosa n* = 78 (82%)	Cell wall synthesis inhibitors	Penicillins (e.g., amoxicillin, ampicillin)	7	13 (346)	4.44
		Cephalosporins (e.g., cephalexin, cefazolin, cefuroxime, cefotaxime, ceftriaxone, cefepime)	7	16 (437)	5.60
		Carbapenems (e.g., meropenem, imipenem)	6	8 (206)	2.64
		Monobactams (e.g. aztreonam)	5	7 (187)	2.40
		Glycopeptide: (e.g., vancomycin)	0	0 (0)	0.00
	Cell membrane disruptors	Polymyxins (e.g., colistin)	1	1 (8)	0.10
		Lipopeptides (e.g., daptomycin)	0	0 (0)	0.00
	DNA/RNA synthesis inhibitors	Fluoroquinolones (e.g., ciprofloxacin, levofloxacin, ofloxacin)	7	16 (475)	6.09
		Rifamycins (e.g., rifampicin)	5	7 (250)	3.21
	Protein synthesis inhibitors	Aminocyclitols (e.g., spectinomycin, tiamulin)	2	2 (25)	0.32
		Aminoglycosides (e.g., gentamycin, amikacin, kanamycin)	9	15 (426)	5.46
		Macrolides (e.g., erythromycin, azithromycin)	13	28 (872)	11.18
		Lincosamides (e.g., clindamycin, lincomycin)	4	8 (158)	2.03
		Tetracyclines (e.g., minocycline, tigecycline)	10	22 (712)	9.13
		Glycylcycline (e.g., tigecycline)	0	0 (0)	0.00
		Amphenicols (e.g., chloramphenicol, thiamphenicol)	10	25 (805)	10.32
	Antimetabolites	Trimethoprim derivatives (e.g., trimethoprim)	8	19 (666)	8.54
		Sulfonamides (e.g., sulfamethoxazole, sulfadiazine)	5	8 (261)	3.35
*Staphylococcus aureus n* = 68 (72%)	Cell wall synthesis inhibitors	Penicillins (e.g., amoxicillin, ampicillin)	7	14 (210)	3.09
		Cephalosporins (e.g., cephalexin, cefazolin, cefuroxime, cefotaxime, ceftriaxone, cefepime)	7	15 (222)	3.26
		Carbapenems (e.g., meropenem, imipenem)	5	7 (101)	1.49
		Monobactams (e.g., aztreonam)	6	7 (192)	2.82
		Glycopeptide: (e.g., vancomycin)	0	0 (0)	0
	Cell membrane disruptors	Polymyxins (e.g., colistin)	0	0 (0)	0
		Lipopeptides (e.g., daptomycin)	0	0 (0)	0
	DNA/RNA synthesis inhibitors	Fluoroquinolones (e.g., ciprofloxacin, levofloxacin, ofloxacin)	9	18 (254)	3.74
		Rifamycins (e.g., rifampicin)	6	4 (77)	1.13
	Protein synthesis inhibitors	Aminocyclitols (e.g., spectinomycin, tiamulin)	1	1 (26)	0.01
		Aminoglycosides (e.g., gentamycin, amikacin, kanamycin)	5	15 (223)	3.28
		Macrolides (e.g., erythromycin, azithromycin)	9	16 (234)	3.44
		Lincosamides (e.g., clindamycin, lincomycin)	4	9 (129)	1.91
		Tetracyclines (e.g., minocycline, tigecycline)	13	25 (315)	4.63
		Glycylcycline (e.g., tigecycline)	1	1 (8)	0.12
		Amphenicols (e.g., chloramphenicol, thiamphenicol)	10	25 (312)	4.59
	Antimetabolites	Trimethoprim derivatives (e.g., trimethoprim)	8	19 (262)	3.85
		Sulfonamides (e.g., sulfamethoxazole, sulfadiazine)	4	7 (88)	1.29
*Acinetobacter baumannii n* = 42 (44%)	Cell wall synthesis inhibitors	Penicillins (e.g., amoxicillin, ampicillin)	5	8 (95)	2.26
		Cephalosporins (e.g., cephalexin, cefazolin, cefuroxime, cefotaxime, ceftriaxone, cefepime)	5	10 (115)	2.74
		Carbapenems (e.g., meropenem, imipenem)	4	4 (32)	0.76
		Monobactams (e.g., aztreonam)	4	4 (48)	1.14
		Glycopeptide: (e.g., vancomycin)	0	0 (0)	0.00
	Cell membrane disruptors	Polymyxins (e.g., colistin)	0	0 (0)	0.00
		Lipopeptides (e.g., daptomycin)	0	0 (0)	0.00
	DNA/RNA synthesis inhibitors	Fluoroquinolones (e.g., ciprofloxacin, levofloxacin, ofloxacin)	4	7 (68)	1.62
		Rifamycins (e.g. rifampicin)	0	0 (0)	0.00
	Protein synthesis inhibitors	Aminocyclitols (e.g., spectinomycin, tiamulin)	1	1 (20)	0.48
		Aminoglycosides (e.g., gentamycin, amikacin, kanamycin)	4	9 (102)	2.43
		Macrolides (e.g., erythromycin, azithromycin)	2	2 (44)	1.05
		Lincosamides (e.g., clindamycin, lincomycin)	4	9 (92)	2.19
		Tetracyclines (e.g., minocycline, tigecycline)	7	10 (81)	1.93
		Glycylcycline (e.g., tigecycline)	1	1 (5)	0.12
		Amphenicols (e.g., chloramphenicol, thiamphenicol)	5	7 (49)	1.17
	Antimetabolites	Trimethoprim derivatives (e.g., trimethoprim)	2	3 (38)	0.90
		Sulfonamides (e.g., sulfamethoxazole, sulfadiazine)	1	1 (5)	0.12
*Escherichia coli n* = 26 (27%)	Cell wall synthesis inhibitors	Penicillins (e.g., amoxicillin, ampicillin)	5	15 (78)	3.0
		Cephalosporins (e.g., cephalexin, cefazolin, cefuroxime, cefotaxime, ceftriaxone, cefepime)	6	18 (93)	3.57
		Carbapenems (e.g., meropenem, imipenem)	3	4 (16)	1.08
		Monobactams (e.g., aztreonam)	3	11 (44)	1.69
		Glycopeptide: (e.g., vancomycin)	0	0 (0)	0.00
	Cell membrane disruptors	Polymyxins (e.g., colistin)	0	0 (0)	0.00
		Lipopeptides (e.g., daptomycin)	0	0 (0)	0.00
	DNA/RNA synthesis inhibitors	Fluoroquinolones (e.g., ciprofloxacin, levofloxacin, ofloxacin)	8	14 (70)	2.69
		Rifamycins (e.g., rifampicin)	7	4 (17)	0.65
	Protein synthesis inhibitors	Aminocyclitols (e.g., spectinomycin, tiamulin)	1	1 (14)	0.54
		Aminoglycosides (e.g., gentamycin, amikacin, kanamycin)	10	3 (73)	2.81
		Macrolides (e.g., erythromycin, azithromycin)	8	16 (128)	4.92
		Lincosamides (e.g., clindamycin, lincomycin)	7	12 (71)	2.73
		Tetracyclines (e.g., minocycline, tigecycline)	13	18 (85)	3.27
		Glycylcycline (e.g., tigecycline)	3	4 (16)	0.62
		Amphenicols (e.g., chloramphenicol, thiamphenicol)	8	13 (57)	2.19
	Antimetabolites	Trimethoprim derivatives (e.g., trimethoprim)	5	6 (35)	1.35
		Sulfonamides (e.g., sulfamethoxazole, sulfadiazine)	2	2 (7)	0.27
*Streptococcus pneumoniae n* = 25 (26%)	Cell wall synthesis inhibitors	Penicillins (e.g., amoxicillin, ampicillin)	5	14 (83)	3.32
		Cephalosporins (e.g., cephalexin, cefazolin, cefuroxime, cefotaxime, ceftriaxone, cefepime)	5	12 (77)	3.08
		Carbapenems (e.g., meropenem, imipenem)	4	6 (26)	1.04
		Monobactams (e.g., aztreonam)	4	7 (30)	1.20
		Glycopeptide: (e.g., vancomycin)	0	0 (0)	0
	Cell membrane disruptors	Polymyxins (e.g., colistin)	0	0 (0)	0
		Lipopeptides (e.g., daptomycin)	0	0(0)	0
	DNA/RNA synthesis inhibitors	Fluoroquinolones (e.g., ciprofloxacin, levofloxacin, ofloxacin)	6	11 (52)	2.08
		Rifamycins (e.g., rifampicin)	3	3 (9)	0.36
	Protein synthesis inhibitors	Aminocyclitols (e.g., spectinomycin, tiamulin)	1	1 (14)	0.56
		Aminoglycosides (e.g., gentamycin, amikacin, kanamycin)	5	8 (69)	2.76
		Macrolides (e.g., erythromycin, azithromycin)	10	19 (166)	6.64
		Lincosamides (e.g., clindamycin, lincomycin)	5	10 (82)	3.28
		Tetracyclines (e.g., minocycline, tigecycline)	7	13 (69)	2.76
		Glycylcycline (e.g., tigecycline)	0	0	0
		Amphenicols (e.g., chloramphenicol, thiamphenicol)	6	10 (43)	1.72
	Antimetabolites	Trimethoprim derivatives (e.g., trimethoprim)	6	6 (34)	1.36
		Sulfonamides (e.g., sulfamethoxazole, sulfadiazine)	2	2 (6)	0.24
*Enterobacter* spp. *n* = 24 (25%)	Cell wall synthesis inhibitors	Penicillins (e.g., amoxicillin, ampicillin)	4	15 (81)	3.38
		Cephalosporins (e.g., cephalexin, cefazolin, cefuroxime, cefotaxime, ceftriaxone, cefepime)	5	17 (88)	3.67
		Carbapenems (e.g., meropenem, imipenem)	2	3 (11)	0.46
		Monobactams (e.g., aztreonam)	3	10 (39)	1.63
		Glycopeptide: (e.g., vancomycin)	0	0 (0)	0.00
	Cell membrane disruptors	Polymyxins (e.g., colistin)	0	0 (0)	0.00
		Lipopeptides (e.g., daptomycin)	0	0 (0)	0.00
	DNA/RNA synthesis inhibitors	Fluoroquinolones (e.g., ciprofloxacin, levofloxacin, ofloxacin)	9	14 (68)	2.83
		Rifamycins (e.g., rifampicin)	3	4 (14)	0.58
	Protein synthesis inhibitors	Aminocyclitols (e.g., spectinomycin, tiamulin)	1	1 (11)	0.46
		Aminoglycosides (e.g., gentamycin, amikacin, kanamycin)	3	7 (52)	2.17
		Macrolides (e.g., erythromycin, azithromycin)	9	17 (122)	5.08
		Lincosamides (e.g., clindamycin, lincomycin)	6	12 (76)	3.17
		Tetracyclines (e.g., minocycline, tigecycline)	8	15 (70)	2.92
		Glycylcycline (e.g., tigecycline)	3	4 (16)	0.67
		Amphenicols (e.g., chloramphenicol, thiamphenicol)	6	12 (50)	2.08
	Antimetabolites	Trimethoprim derivatives (e.g., trimethoprim)	4	5 (32)	1.33
		Sulfonamides (e.g., sulfamethoxazole, sulfadiazine)	1	1 (3)	0.13
*Group B Streptococcus n* = 13 (14%)	Cell wall synthesis inhibitors	Penicillins (e.g., amoxicillin, ampicillin)	5	30 (67)	0.19
		Cephalosporins (e.g., cephalexin, cefazolin, cefuroxime, cefotaxime, ceftriaxone, cefepime)	6	33 (75)	5.77
		Carbapenems (e.g., meropenem, imipenem)	4	9 (20)	1.54
		Monobactams (e.g., aztreonam)	5	13 (26)	2
		Glycopeptide: (e.g., vancomycin)	1	1 (1)	0.08
	Cell membrane disruptors	Polymyxins (e.g., colistin)	0	0	0
		Lipopeptides (e.g., daptomycin)	0	0	0
	DNA/RNA synthesis inhibitors	Fluoroquinolones (e.g., ciprofloxacin, levofloxacin, ofloxacin)	4	7 (9)	0.69
		Rifamycins (e.g., rifampicin)	5	7 (10)	0.77
	Protein synthesis inhibitors	Aminocyclitols (e.g., spectinomycin, tiamulin)	2	2 (7)	0.54
		Aminoglycosides (e.g., gentamycin, amikacin, kanamycin)	4	19 (44)	3.38
		Macrolides (e.g., erythromycin, azithromycin)	10	19 (73)	5.62
		Lincosamides (e.g., clindamycin, lincomycin)	8	16 (51)	3.92
		Tetracyclines (e.g., minocycline, tigecycline)	11	25 (47)	3.62
		Glycylcycline (e.g., tigecycline)	2	2 (2)	0.15
		Amphenicols (e.g., chloramphenicol, thiamphenicol)	12	12 (25)	1.92
	Antimetabolites	Trimethoprim derivatives (e.g., trimethoprim)	8	9 (24)	1.84
		Sulfonamides (e.g., sulfamethoxazole, sulfadiazine)	4	4 (5)	0.38
*Enterococcus faecium n* = 10 (11%)	Cell wall synthesis inhibitors	Penicillins (e.g., amoxicillin, ampicillin)	4	22 (45)	4.5
		Cephalosporins (e.g., cephalexin, cefazolin, cefuroxime, cefotaxime, ceftriaxone, cefepime)	5	22 (46)	4.6
		Carbapenems (e.g., meropenem, imipenem)	6	10 (14)	1.4
		Monobactams (e.g., aztreonam)	5	9 (15)	1.5
		Glycopeptide: (e.g., vancomycin)	1	1 (1)	0.1
	Cell membrane disruptors	Polymyxins (e.g., colistin)	0	0 (0)	0
		Lipopeptides (e.g., daptomycin)	0	0 (0)	0
	DNA/RNA synthesis inhibitors	Fluoroquinolones (e.g., ciprofloxacin, levofloxacin, ofloxacin)	8	21 (35)	3.5
		Rifamycins (e.g., rifampicin)	6	9 (10)	1
	Protein synthesis inhibitors	Aminocyclitols (e.g., spectinomycin, tiamulin)	1	1 (5)	0.5
		Aminoglycosides (e.g., gentamycin, amikacin, kanamycin)	5	17 (40)	4.0
		Macrolides (e.g., erythromycin, azithromycin)	9	19 (54)	5.4
		Lincosamides (e.g., clindamycin, lincomycin)	9	16 (51)	5.1
		Tetracyclines (e.g., minocycline, tigecycline)	9	26 (40)	4.0
		Glycylcycline (e.g., tigecycline)	2	2 (2)	0.2
		Amphenicols (e.g., chloramphenicol, thiamphenicol)	11	16 (23)	2.3
	Antimetabolites	Trimethoprim derivatives (e.g., trimethoprim)	7	12 (22)	2.2
		Sulfonamides (e.g., sulfamethoxazole, sulfadiazine)	5	5 (5)	0.5
*Klebsiella pneumoniae n* = 8 (8%)	Cell wall synthesis inhibitors	Penicillins (e.g., amoxicillin, ampicillin)	8	28 (54)	6.75
		Cephalosporins (e.g., cephalexin, cefazolin, cefuroxime, cefotaxime, ceftriaxone, cefepime)	8	29 (57)	7.13
		Carbapenems (e.g., meropenem, imipenem)	3	6 (6)	0.75
		Monobactams (e.g., aztreonam)	4	14 (31)	3.88
		Glycopeptide: (e.g., vancomycin)	1	1 (1)	0.13
	Cell membrane disruptors	Polymyxins (e.g., colistin)	0	0 (0)	0.00
		Lipopeptides (e.g., daptomycin)	0	0 (0)	0.00
	DNA/RNA synthesis inhibitors	Fluoroquinolones (e.g., ciprofloxacin, levofloxacin, ofloxacin)	15	29 (58)	7.25
		Rifamycins (e.g., rifampicin)	7	14 (18)	2.25
	Protein synthesis inhibitors	Aminocyclitols (e.g., spectinomycin, tiamulin)	1	1 (3)	0.38
		Aminoglycosides (e.g., gentamycin, amikacin, kanamycin)	9	19 (30)	3.75
		Macrolides (e.g., erythromycin, azithromycin)	16	36 (62)	7.75
		Lincosamides (e.g., clindamycin, lincomycin)	9	27 (27)	3.38
		Tetracyclines (e.g., minocycline, tigecycline)	19	43 (71)	8.88
		Glycylcycline (e.g., tigecycline)	3	4 (16)	2.0
		Amphenicols (e.g., chloramphenicol, thiamphenicol)	14	36 (59)	7.38
	Antimetabolites	Trimethoprim derivatives (e.g., trimethoprim)	11	26 (37)	4.63
		Sulfonamides (e.g., sulfamethoxazole, sulfadiazine)	5	8 (9)	1.13
*Enterococcus faecalis n* = 4 (4%)	Cell wall synthesis inhibitors	Penicillins (e.g., amoxicillin, ampicillin)	3	7 (15)	3.75
		Cephalosporins (e.g., cephalexin, cefazolin, cefuroxime, cefotaxime, ceftriaxone, cefepime)	3	7 (15)	3.75
		Carbapenems (e.g., meropenem, imipenem)	2	2 (3)	0.75
		Monobactams (e.g., aztreonam)	3	4 (6)	1.50
		Glycopeptide: (e.g., vancomycin)	0	0	0
	Cell membrane disruptors	Polymyxins (e.g., colistin)	0	0	0
		Lipopeptides (e.g., daptomycin)	0	0	0
	DNA/RNA synthesis inhibitors	Fluoroquinolones (e.g., ciprofloxacin, levofloxacin, ofloxacin)	4	8 (12)	3.0
		Rifamycins (e.g., rifampicin)	3	3 (4)	1
	Protein synthesis inhibitors	Aminocyclitols (e.g., spectinomycin, tiamulin)	1	1 (4)	1
		Aminoglycosides (e.g., gentamycin, amikacin, kanamycin)	4	7 (15)	3.75
		Macrolides (e.g., erythromycin, azithromycin)	6	11 (21)	5.25
		Lincosamides (e.g., clindamycin, lincomycin)	7	12 (22)	5.5
		Tetracyclines (e.g., minocycline, tigecycline)	4	8 (11)	2.75
		Glycylcycline (e.g., tigecycline)	0	0	0
		Amphenicols (e.g., chloramphenicol, thiamphenicol)	4	6 (9)	2.25
	Antimetabolites	Trimethoprim derivatives (e.g., trimethoprim)	5	5 (8)	2
		Sulfonamides (e.g., sulfamethoxazole, sulfadiazine)	1	1 (1)	0.25

### 3.3 Resistome Associated With High Mortality Associated Bacteria on Healthcare Workers Mobile Phones

A total of 262 unique ARGs were identified (total Hits 2475) from the 95 mobile phone swabs (Table 1). Among the samples confirmed to contain gram‐negative bacteria of interest, the dominant resistance signatures were linked to cell wall synthesis inhibitors (penicillins, cephalosporins, carbapenems, and monobactams), fluoroquinolones, aminoglycosides, and tetracyclines. High numbers of resistance mechanisms and gene hits were observed associated with *P. aeruginosa* (e.g., 437 cephalosporin‐related hits, 475 fluoroquinolone‐related hits, and 426 aminoglycoside‐related hits), reflecting the potential for broad intrinsic and acquired resistance properties of this species. The mechanisms commonly identified displayed a broad and multilayered resistome comprising of mechanisms related to enzymatic inactivation, permeability barriers, and efflux pumps, which provide increased multidrug resistance. Genes associated with polymyxin resistance (*pmrB*; Hits: 8) were restricted to *P. aeruginosa*. In addition to *P. aeruginosa*, mechanisms and associated genes conferring carbapenem resistance, an antibiotic class commonly regarded as a “last line therapy” for gram‐negative infections, such as efflux systems (e.g., MexAB‐OprM, MexPQ‐OpmE) and β‐lactamase enzymatic inactivation (*blaOXA*, *blaPDC*), were also co‐located on mobile phone samples containing *E. coli, K. pneumoniae, A. baumannii*, and *Enterobacter* spp.

With respect to gram‐positive bacteria associated with high global mortality, the dominant resistance signatures of greatest clinical relevance were associated with β‐lactams, macrolides, lincosamides, and glycopeptides. Additionally, *S. aureus* methicillin resistance determinants (mec family genes) were co‐located on 22% of mobile phones alongside genes associated with mecA upregulation via transcriptional and regulatory mechanisms (e.g., *mecl*, *mecR1*). However, SSCmec‐specific cassette chromosome recombinase components (*ccrA*, *ccrB*, *ccrC*, and *orfX* junctions) were not identified in the data set. With regards to *S. pneumoniae*, alongside penicillin‐binding‐protein genes (e.g., PBP2b: Hits 11), macrolide resistance was prominent (166 hits), consistent with the clinical challenge of macrolide‐resistant pneumococcal infections. Numerous genes co‐located with *A. baumannii*, associated with resistance nodulation division efflux pumps (e.g., AdelJK, AdeFGH) were also identified. However, tripartite sections, specifically *adeK* and *adeG*, were not identified. In *Group B Streptococcus*, resistance determinants were identified against β‐lactams (penicillins and cephalosporins) and macrolides (73 hits), the two most clinically relevant classes given their role in first‐line and penicillin‐allergy management, respectively. Notably, whilst a vancomycin resistance gene (*vanXY*) was co‐located with *E. faecium*, the *vanA* and *vanB* operons associated with clinically important *E. faecium* (*VREfm*) were not identified. However, genes associated with resistance were identified for tetracyclines, amphenicols, and aminoglycosides across species, which, whilst not routinely used as frontline agents for gram‐positive infections or utilized only in combination with other antibiotic classes, highlight the potential for multidrug resistance organisms to develop with the potential to complicate clinical treatment.

### 3.4 Virulome Associated With Healthcare Worker Mobile Phones

A virulome representing 448 unique VFGs was identified across the 10 bacterial species investigated, 409 of which were co‐located with bacteria known to be associated with the specific genes. Notably, *P. aeruginosa* (Hits: 1481; average number of VFGs per phone: 20.29) and *S. aureus* (Hits: 5779; average number of VFGs per mobile phone: 74.09) exhibited the highest prevalence and diversity of associated VFGs. The relative percentage of VFGs being co‐located with bacteria varied considerably from 85% to 100% in *E. faecalis*, *K. pneumoniae*, *S. aureus*, and *P. aeruginosa*. No VFGs were identified for *A. baumannii*. Across all samples, the identified VFGs encompassed a wide range of mechanisms which, when expressed, may confer survival advantages as well as increased pathogenicity.

Unique VFGs such as *qacC*, *qacEdelta1*, and *qacE*, which encode mechanisms that reduce susceptibility to quaternary ammonium disinfectants, were co‐located on 56, 25, and 2 mobile phones, respectively, alongside *S. aureus*, *P. aeruginosa*, and *E. coli*. VFGs identified to be co‐located with *P. aeruginosa* include *pilN* (type IV pilus assembly), *xcpW* (type II secretion), and the alginate pathway genes *algD*, *algE*, and *algZ*, conveying qualities associated with increased secretion, surface motility, and alginate‐mediated biofilm formation that enhance environmental persistence and immune evasion. Genes associated with hemolysis such as *hla* (Hits: 2) and *hlb* (Hits: 5) were identified to be co‐located with *S. aureus* in addition to *plcH* (Hits: 39) with *P. aeruginosa*. Moreover, the *tsst* gene (Hits: 2), a superantigen associated with toxic shock syndrome, was co‐located with *S. aureus*. Similarly, the gene *xcpW*, a component of the Type II secretion system associated with *P. aeruginosa* contributes, when expressed, to toxin secretion and host tissue damage.

In addition to environmental persistence and toxin production, several bacterial species were co‐located with VFGs that enhance host colonization and increase pathogenic potential. Genes such as *pilN* and *psaA*, co‐located with *P. aeruginosa* and *S. pneumoniae,* respectively, facilitate motility and adhesion to host tissues, a critical step in the initiation of infection. Similarly, *S. pneumoniae* was also co‐located with *nanB* and *cbpE* supporting increased cellular adhesion and immune evasion.

Together, these genes represent multiple virulence strategies that not only facilitate initial infection and colonization, but also support immune evasion and tissue invasion, thereby increasing the likelihood of clinically important infections in hospital environments. The direct metagenomic sequencing approach prevents robust analysis of the degree to which horizontal gene transfer (HGT) of these genes occurs on mobile phones within healthcare settings. However, the co‐location of these genes alongside genes encoding transposons (*tnpA*), plasmid conjugation machinery (*trbK*, *tral*, *traJ*, *traC‐4*), and plasmid mobilization and replication genes (*mobB*, *repB*) provides evidence for the possibility for HGT to occur.

### 3.5 Bacteriophages Associated With Target Bacteria

A total of 314 unique bacteriophages were identified across all mobile phone samples (2988 total hits), with a mean of 67.7 phages per device (range, 4–235; Table 2). Bacteria‐bacteriophage co‐locations were most frequent for *S. aureus* (84 unique phages; 1104 hits), *P. aeruginosa* (53 unique phages; 489 hits), and *S. pneumoniae* (45 unique phages; 692 hits) (Table 3). Several abundant staphylococcal lineages detected in the data set, such as StB20‐like, StB12, and IME‐SA4, are temperate and have been linked to lysogeny with carriage of virulence regulators and toxins, such as PVL‐converting phages encoding Panton–Valentine leukocidin, φETA family phages encoding exfoliative toxin A, and cdt‐converting phages encoding cytolethal distending toxin. In Enterobacteriaceae, Shiga toxin–converting (Stx) phages provide an analogous paradigm of phage‐borne toxigenic augmentation. Phages associated with *P. aeruginosa* (richness 53; 489 hits) identified within samples are implicated in environmental remodeling, including the promotion of biofilm formation and modulation of secretion systems through filamentous *Pf‐*like phages, temperate lineages such as D3/D3112/DMS3, and *φCTX‐*like, with downstream effects on persistence and antibiotic tolerance. Staphylococcal prophages frequently encode immune‐modulatory factors that attenuate neutrophil and complement activity, and phage activity within *P. aeruginosa* biofilms has likewise been associated with dampening of host responses, facilitating chronic colonization. Finally, streptococcal and pneumococcal phages (e.g., *phage 20617* and *φARI0131‐2*) have been implicated in HGT of capsule/adhesin and other virulence determinants. Collectively, baceteriophages associated with toxin carriage, environmental remodeling, immune evasion, and gene transfers indicate that mobile phones may harbor a substantial phage reservoir with the potential to modulate virulence and treatment response in bacteria relevant to HAIs.

**Table 2:** Virulence factor genes associated with bacteria of interest.

Microorganism, number of phones (Relative percentage)	Number of unique virulence factor genes	Number of unique VFGs co‐located with target bacteria	Total number of hits	Average number of VFGs per mobile phone	Percentage of mobile phones with VFGs and bacteria co‐located
*Pseudomonas aeruginosa n* = 78 (82%)	158	158	5779	74.09	87.18%
*Staphylococcus aureus n* = 68 (72%)	179	179	1481	20.29	98.53%
*Acinetobacter baumannii n* = 42 (44%)	0	0	0	0	0%
*Escherichia coli n* = 26 (27%)	9	6	24	0.92	42.31%
*Streptococcus pneumoniae n* = 25 (26%)	5	5	19	0.76	52%
*Enterobacter* spp. *n* = 24 (25%)	60	39	35	1.46	62.5%
*Group B Streptococcus n* = 13 (14%)	1	1	1	0.10	10%
*Enterococcus faecium n* = 10 (11%)	9	6	15	1.5	80%
*Klebsiella pneumoniae n* = 8 (8%)	20	10	26	3.25	100%
*Enterococcus faecalis n* = 4 (4%)	7	5	12	3.0	100%

**Table 3:** Bacteriophages associated with bacteria associated with high mortality on mobile phones.

**Microorganism, number of phones (Relative percentage)**	**Number of unique phages**	**Number of unique phages co‐located with target bacteria**	**Total number of hits**	**Average number of phages per mobile phone**	**Percentage of mobile phones with phages and bacteria co‐located**
*Pseudomonas aeruginosa n* = 78 (82%)	57	53	489	5.89	87.18%
*Staphylococcus aureus n* = 68 (72%)	85	84	1104	16.24	97.06%
*Acinetobacter baumannii n* = 42 (44%)	8	6	85	1.81	66.67%
*Escherichia coli n* = 26 (27%)	27	22	106	4.08	84.62%
*Streptococcus pneumoniae n* = 25 (26%)	46	45	692	27.68	96%
*Enterobacter* spp. *n* = 24 (25%)	25	22	103	3.55	66.67%
*Group B Streptococcus n* = 13 (14%)	46	43	392	30.15	100%
*Enterococcus faecium n* = 10 (11%)	7	3	5	0.50	40%
*Klebsiella pneumoniae n* = 8 (8%)	6	2	2	0.25	12.50%
*Enterococcus faecalis n* = 4 (4%)	7	5	10	2.50	100%

## 4 Discussion

Antimicrobial resistance has been highlighted as a global threat responsible for the deaths of millions of people and resulting in trillions of dollars of healthcare‐related economic expenditure (Jonas et al. 2017). Around the world, researchers and clinicians continue to collaborate to prevent HAIs, identify and address the drivers of increasing AMR, and develop new strategies for treating antibiotic resistant infections. The results of this study highlight that antibiotic resistant bacteria known to be associated with high mortality are commonly co‐located on the mobile phones of healthcare workers. As a result, mobile phones may not only act as reservoirs for pathogens but may also act as platforms which enable HGT accelerating AMR development.

### 4.1 Clinical Implications of Mobile Phones as Fomites in Hospital Settings

Our findings have immediate clinical implications. Healthcare workers' phones were found to frequently harbor high‐mortality HAI‐associated taxa that were co‐located with clinically relevant resistance and virulence gene determinants. This finding indicates the presence of bacteria that, if contributing to a HAI, may result in increased pathogenicity and decreased treatability. Across mobile phone samples, the data showed that ARG profiles were consistent with front‐line therapeutic failure in gram‐negatives (e.g., β‐lactamases including OXA/PDC families and robust efflux systems such as MexAB‐OprM/MexPQ‐OpmE) and gram‐positives (e.g., mec‐regulated methicillin resistance in *S. aureus*), alongside polymyxin pathway regulators (*pmrB*) in *P. aeruginosa* (Moskowitz et al. 2012; Tsutsumi et al. 2019; Wielders et al. 2002). Virulence modules that promote persistence and host damage were common. For example, biofilm and secretion genes were co‐located with *P. aeruginosa* (such as *algD*/*algE*/*algZ*, *xcpW*), hemolysins, a superantigen (*hla*/*hlb*, *tsst*) with *S. aureus*, and adhesion/immune‐evasion factors with *S. pneumoniae* (*nanB*, *cbpE*) (Vandenesch et al. 2012; Schlievert et al. 2008; Schinner et al. 2020; Rajabi et al. 2022; Xu et al. 2008). Notably, disinfectant‐tolerance genes (*qacC*, *qacEΔ1*) were co‐located with major pathogens, raising concern that routine quaternary‐ammonium based cleaning may incompletely mitigate risk, particularly in the context of biofilm (Wassenaar et al. 2016). The phage reservoir detected on phones further heightens clinical concern: temperate staphylococcal and enterobacterial phages known to carry toxins (e.g., PVL‐, ETA‐, and cdt‐converting phages; Shiga toxin–converting phages) and Pf‐like/Pseudomonas temperate lineages that enhance biofilm and antibiotic tolerance provide mechanisms which, when expressed, modulate virulence and treatment response in situ. Whilst markers consistent with intrinsic VanC‐type resistance (*vanC* and *vanXYC* genes) were detected within *Enterococcus gallinarum*. No *vanA*, *vanB* operons (aka vanHAX clusters) were identified indicative of bacteria associated with clinical outbreaks of VRE relating to vancomycin resistant *E. faecalis* or *faecium* (van Hal et al. 2017, 2018). However, when taken together, this metagenomic data positions mobile phones as high‐touch fomites that can contaminate patients and staff with pathogenic microbes, undermine environmental hygiene controls, and complicate therapy via contributing to HGT.

### 4.2 Potential for Horizontal Genetic Transfer to Occur on Mobile Phones

HGT in clinical environments is mediated not only by the classical mechanisms of transformation, conjugation, and transduction, but also by a diverse repertoire of mobile genetic elements (plasmids, integrons, transposons/insertion sequences, integrative and conjugative elements, and prophages) within an environment with the potential to package, mobilize, and stabilize resistance and virulence cargo (Burmeister 2015). In the present data set, the recurrent detection of class 1/3 integron components (*intI1*/*intI3*, *qacEΔ1*), transposition functions (with genes such as *tnpA* and *tniA*–*C*), and conjugation modules (*tra*/*trb*/*incC1*, *mob*/*rep*, *korA*/*korB*/*korC*) alongside high‐mortality pathogens suggests a genomic context permissive for co‐selection and mobilization of ARGs and VFGs (Virolle et al. 2020).

Conjugation is the main hospital‐scale route for ARG dissemination and is driven by plasmid‐encoded systems (Cabezón et al. 2017). In our data set, we repeatedly observed four complementary module types. First, the conjugative transfer apparatus consisting of type IV secretion systems and mating pair formation tra/trb clusters that build the conjugative pilus and trans‐envelope channel for DNA export (Cabezón et al. 2017). Second, mobilization/Dtr genes which enable the relaxase (e.g., *tral*/*mobB*) to nick and prepare plasmid DNA for export. Third, replication and maintenance genes such as *repB* and the IncC1 replicon marker that contribute to plasmid stability, and finally IncP‐1 regulators (*korA/korB/korC*) that tune expression of replication and transfer loci to balance fitness costs. We also detected transposition machinery (*tnpA*, *tnpR*) and class 1 integron hallmarks (qacEΔ1 and, in some samples, intI1), which function as a cassette system that commonly carries aminoglycoside *aadA* variants (Chang et al. 2007; Deng et al. 2015). Taken together, this architecture is consistent with conjugation‐competent platforms capable of (i) plasmid persistence, (ii) cassette acquisition/turnover, and (iii) interspecies transfer under selection. Because phones are repeatedly exposed to disinfectants, the frequent recovery of quaternary ammonium compound determinants (e.g., qacC in *S. aureus* and *qacEΔ1* with Enterobacterales/*P. aeruginosa*) suggests a plausible route for co‐selection of integron‐bearing plasmids during routine cleaning.

Beyond simple uptake of extracellular DNA (eDNA), effective transformation requires (i) competent hosts, (ii) protected/abundant DNA, and (iii) recombination (Burmeister 2015; Chen et al. 2005). Mobile phones are repeatedly inoculated and containing niches (e.g., in cases/crevices) in which biofilm matrices could provide protection to eDNA (Madsen et al. 2012). The co‐detection of biofilm/alginate genes in *P. aeruginosa* (e.g., *algD/algE/algZ*, type II secretion *xcp* genes) and staphylococcal biofilm locus (*ica*) is consistent with microenvironments with the ability to stabilize eDNA increasing transformation probabilities (Madsen et al. 2012). Naturally transformable healthcare‐associated bacteria (e.g., *Acinetobacter* spp., *S. pneumoniae*) were present; in such taxa, biofilm‐associated competence can promote uptake of ARG/VFG fragments (Madsen et al. 2012).

Phage‐driven HGT encompasses generalized, specialized, and lateral transduction, as well as prophage cargo (“lysogenic conversion”) (Borodovich et al. 2022). The mobile phones harbored abundant temperate staphylococcal phages (e.g., StB20‐like, StB12, IME‐SA4) linked to virulence cargo (PVL‐, φETA‐, and cdt‐converting paradigms) and pneumococcal/streptococcal phages implicated in capsule/adhesin exchange. For *P. aeruginosa*, Pf‐like filamentous phages and temperate lineages (e.g., D3/D3112/DMS3, φCTX‐like) can modulate biofilms, quorum, and secretion systems that both stabilize DNA and increase transduction opportunities (Secor et al. 2015). Environmental stressors relevant to mobile phones, such as UV exposure, desiccation, and sub‐MIC antibiotics/disinfectants, have the potential to induce prophages, transiently amplifying generalized transduction (Liao et al. 2024; Bailey et al. 2024). While read‐based signals cannot distinguish active transduction events, the co‐location of phages with their bacterial hosts plus ARG/VFG signatures is consistent with a transduction‐capable virome on these surfaces.

### 4.3 Sanitization of Mobile Phones in Healthcare Settings

The presence of ARGs, VFGs, and bacteriophages both within this study and in other culture‐based investigations on healthcare worker mobile phones underscores the limitations of current disinfection practices. Although alcohol‐based disinfectants, particularly 70% isopropyl alcohol, are widely recommended, their use remains inconsistent in clinical settings. These agents effectively reduce viable bacterial counts but are less effective in degrading biofilms and eDNA, which may persist on surfaces following cell lysis (von Hertwig et al. 2023; Luther et al. 2015). Biofilms are notoriously resistant to many conventional disinfectants due to their extracellular polymeric substance matrix (Luther et al. 2015; Di Martino 2018). In this study, the identification of genes such as *algR*, *icaB*, and *pilY*1 suggests that bacteria on mobile phones may exist in a biofilm phenotype, which can shield embedded cells and eDNA from alcohol‐based wipes and surface cleaners. Given the co‐location of genes associated with transformation and transposition (*tnpA*) in this study, retained eDNA may act as a substrate for HGT, particularly transformation. The effectiveness of quaternary ammonium disinfectants may be undermined by resistance genes such as *qacC*, which confer reduced susceptibility and frequently co‐localize with clinically relevant bacteria. Exposure to sublethal sanitization products such as quaternary ammonium disinfectants can impose selective pressure and may co‐select mobile genetic elements carrying additional antimicrobial‐resistance determinants. As a result, appropriate contact time and mechanical removal is required, especially with respect to established biofilms. In contrast, chlorine‐based wipes are widely used in healthcare and can be effective against biofilms under appropriate conditions. However, they are also corrosive to many materials, including phone surfaces. This highlights the need for sanitation methods capable of penetrating biofilm matrices, without damaging individuals personal mobile phone devices.

**Figure 1:**
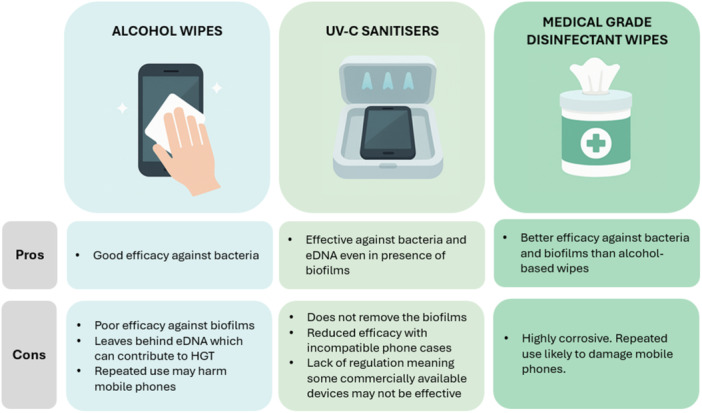
Pros and cons of mobile phone sanitization strategies.

Ultraviolet‐C (UV‐C) phone disinfection has been proposed as a contactless alternative capable of inactivating bacteria and viruses through DNA damage, with several studies demonstrating > 99% microbial reduction on mobile phone surfaces under controlled conditions (Olsen, Goldsworthy, et al. 2023; Ma et al. 2022; Palma et al. 2025). The use of UVC to clean phones in hospitals is warranted when sourced from manufacturers able to demonstrate their devices are robust in germicidal efficacy within appropriate timeframes (10–20 s) whilst being enclosed for operational safety (Figure 1). However, UV‐C exposure is sensitive to dosage, duration, and surface geometry associated with varying devices and protective case configurations, and may not eliminate eDNA (Torkzadeh et al. 2021). The UV‐C disinfection industry has historically lacked stringent regulatory oversight, allowing the proliferation of devices with unverified germicidal efficacy claims and, in some cases, inadequate safety controls. As a result, some commercially available UV‐C units may provide insufficient microbial inactivation or pose unintended risks to users through improper shielding or emissions outside the effective UV‐C spectrum. While genes related to UV‐C tolerance, such as *recA* or *uvrA*, are known to contribute to bacterial survival following UV exposure, the presence of these have not been investigated to date on mobile phones (Phannarangsee et al. 2024; Knobling et al. 2023; Kiran and Patil 2022). Furthermore, suboptimal UV‐C dosing not only risks incomplete microbial inactivation but may induce stress responses in bacteria such as *Bacillus* sp., increasing their environmental resilience and inducing sporulation (Shrestha et al. 2025). This raises additional concerns for device efficacy, particularly in clinical settings. Whilst common clinical fungal pathogens (e.g., *Aspergillus* spp.) demonstrate decreased sporulation following UV exposure, caution must be undertaken to ensure no unintended consequences of ineffective UV‐C use arise.

### 4.4 Limitations

Samples analyzed within this study utilized DNA recovered from phone surfaces at single time points from various studies, located within different settings and countries with very different environmental microbiome profiles. The presence of MGEs in additional settings is likely to also be different necessitating caution when extrapolating these results beyond the current settings. Environmental metagenomics detects nucleic acids irrespective of viability or expression. Additionally, eDNA and DNA from nonviable or stressed cells (e.g., after partial disinfection) can persist and be detected. Consequently, gene presence does not equate to phenotypic resistance, virulence expression, or transmission risk. Most data sets were generated by direct sequencing of swabs using short reads. While appropriate for breadth, the sequencing depths achieved (dozens of Mbp per sample) limit sensitivity for low‐abundance organisms and constrain de novo assembly. Without long‐read or proximity‐linking methods, we cannot reliably place ARGs, VFGs, or phage cargo on specific chromosomal backgrounds or mobile genetic elements, nor resolve operons, cassettes, or integrons. As a result, reference to co‐location within this study refers only to co‐detection within the same mobile phone sample rather that the physical linkage on the same contig/plasmid or within the same microbial cell. The ≥ 10% annotation threshold improves specificity but may still over‐ or under‐represent true host–gene associations, especially for genes with broad host ranges (e.g., qac family, class 1 integron components). Finally, taxonomic, ARG, VFG, and phage cells rely on reference databases and k‐mer–based classifiers. As a result, novel variants, recombined phage modules, and under sampled taxa may be missed or misassigned.

### 4.5 Future Directions Focusing on Intervention Implementation

Decreased microbial load resulting from increased cleaning of environmental surfaces has been shown to decrease HAIs (Browne et al. 2024). To translate the current findings into effective infection prevention strategies, future research must focus on high‐priority clinical settings such as intensive care units and surgical theaters, where pathogen transmission risks are highest. Importantly, future studies should integrate both clinical microbiology with implementation science to ensure that proposed interventions are not only evidence‐based but also feasible, scalable, and cost‐effective as well as acceptable by healthcare professionals and consumers in real‐world clinical contexts. Behavioral frameworks such as the COM‐B model (Capability, Opportunity, Motivation – Behavior) and the Theoretical Domains Framework (TDF) offer structured approaches to identifying barriers and facilitators which assist in highlighting considerations for future large scale hospital interventions (Table 4) (Greene and Wilson 2022; Dyson and Cowdell 2021). For example, the implementation of UV‐C sanitization devices must consider hospital‐specific workflows, cultural norms, and practical constraints such as the diversity of mobile phone cases, which may interfere with efficacy. By embedding such considerations into intervention design from the outset, hospitals can enhance the likelihood of adoption, fidelity, and sustained impact of disinfection protocols aimed at reducing mobile phone‐mediated pathogen transmission. The broad adoption of interventions aimed at tackling risks posed by small fomites such as (but not limited to) mobile phones will also be facilitated by studies demonstrating the cost‐saving nature of these interventions. To reduce HAIs most effectively, mobile phone cleaning should be introduced as part of a broader bundle of interventions targeting high‐touch surfaces (e.g., curtains, bedside tables, bed rails, and monitors) to reduce environmental microbial load.

**Table 4:** Implementation considerations relating to mobile phone cleaning in healthcare settings.

COM‐B domain	TDF domain	Barriers	Facilitators
Capability	Knowledge	Lack of causative evidence of fomites role in HAIs, HGT, and the limitations of certain cleaning methods.	Robust knowledge dissemination strategy transecting both local and public health policies.
	Skills	Lack of understanding of use of UV‐C devices.	Clear visualizations attached to UV‐C devices demonstrating ease of use.
	Memory, attention, and decision processes	Cognitive load associated with clinical demands limits behavior change.	Integrate UV‐C phone hygiene into existing workflows (e.g., during hand hygiene routines).
	Behavioral regulation	Lack of prompts or reminders to clean mobile phones.	Visual cues or automated reminders near sanitizer stations, entrances, and exits of high‐priority areas (e.g., ICU, operating theaters).
Motivation	Beliefs about capabilities	Uncertainty about proper use or efficacy.	Training and signage that build confidence in effective use.
	Social/professional role identity	Perception that phone hygiene is not part of clinical role.	Reframe phone hygiene as integral to infection control responsibilities.
	Beliefs about consequences	Doubts about the value or impact of UV‐C sanitation.	Emphasize evidence linking phone contamination with patient safety risks.
	Emotions	Fear of damage to personal device.	Messaging to clarify that UV‐C is nondamaging to phones and widely used in other hygiene contexts.
	Goals	Competing clinical priorities.	Align UV‐C use with broader patient safety and quality goals.
	Intentions	Low prioritization.	Public commitments or pledges to maintain device hygiene.
	Reinforcement	Lack of feedback or reward.	Include in audit/feedback cycles or quality improvement metrics.
	Optimism/pessimism	Pessimism about compliance by peers.	Visible role‐modeling by senior staff or UV‐C use included in orientation processes
Opportunity	Environmental context and resources	Lack of access to UV‐C stations or inconvenient placement.	Strategically place UV‐C stations in high‐traffic staff areas (e.g., break rooms, hand hygiene points).
	Social influences	Peer apathy or resistance to change.	Social norm‐setting, champions, or team‐based encouragement.

## 5 Conclusion

Metagenomic analysis of 95 mobile phone samples from healthcare workers revealed a high prevalence of bacterial pathogens responsible for the majority of global deaths due to bacterial infections. Notably, several of the WHO priority pathogens were identified, including *S. aureus*, *P. aeruginosa*, *K. pneumoniae*, and *E. coli*. The common presence of clinically relevant pathogens in conjunction with ARGs and VFGs highlights mobile phones as a problematic fomite which harbor bacteria alongside genes associated with increased pathogenicity and decreased treatability. Additionally, the recurrent, cross‐sample detection of mobile genetic elements that underpin HGT indicates that healthcare workers' phones constitute plausible hubs for MGE assembly, maintenance, and exchange. While our environmental metagenomic design cannot prove event‐level transfer, the convergent genomic architecture and selective milieu provide evidence that small objects such as mobile phones in healthcare settings are not passive carriers but act as microhabitats for HGT contributing to the global development of antimicrobial resistance. As a result, sanitization strategies to disinfect mobile phones should be implemented as part of a broader antimicrobial stewardship program with the intention of reducing HAIs and reducing their associated human and economic burden.

## Author Contributions

**Adrian Goldsworthy:** conceptualization, methodology, formal analysis, data curation, project administration, writing – review and editing, writing – original draft. **Matthew Olsen:** conceptualization, investigation, methodology, validation, writing – review and editing, formal analysis. **Nchafatso G. Obonyo:** supervision, writing – review and editing, methodology. **Peter Jones:** writing – review and editing, supervision. **Simon McKirdy:** writing – review and editing, supervision, funding acquisition. **Abiola Senok:** supervision, writing – review and editing, funding acquisition. **Rashed Alghafri:** writing – review and editing, supervision. **Rose Ghemrawi:** writing – review and editing, supervision. **Reem Almheiri:** writing – review and editing, supervision. **Oystein Tronstad:** writing – review and editing, supervision. **Jacky Y. Suen:** writing – review and editing, supervision. **John F. Fraser:** writing – review and editing, supervision. **Lotti Tajouri:** validation, methodology, formal analysis, supervision, writing – review and editing.

## Funding

The authors have nothing to report.

## Ethics Statement

The authors have nothing to report.

## Conflicts of Interest

The authors declare no conflicts of interest.

## Supporting information


**Table S1:** Overview of samples included in this metagenomics metanalysis.

## Data Availability

Data are available within the Supporting File.
