# Supplementary material for: Hospital‐Associated Antimicrobial Resistant Bacteria on 95 Mobile Phones: An International Metagenomic “Phonome” Analysis
Source: Microbiologyopen. 2026 Jun 18;15(3):e70321. doi: 10.1002/mbo3.70321 (PMC13280219; doi:10.1002/mbo3.70321)
Supplement: Supplementary file 1 — Table S1: Overview of samples included in this metagenomics metanalysis. [file MBO3-15-e70321-s001.docx]

**Supplementary Table 1.** Overview of samples included in this metagenomics metanalysis.

| **Sample number** | **Source code** | **Country** | **Location** | **Setting** | **Sequencing Technique** | **SRA numbers** |
| --- | --- | --- | --- | --- | --- | --- |
| Sample 1 | NS231-02 | Australia | Hospital | Paediatric ward (general) | Direct NGS | [**SRX14961713**](https://www.ncbi.nlm.nih.gov/sra/SRX14961713%5Baccn%5D) |
| Sample 2 | NS231-03 | Australia | Hospital | Paediatric ward (general) | Direct NGS | [**SRX14961714**](https://www.ncbi.nlm.nih.gov/sra/SRX14961714%5Baccn%5D) |
| Sample 3 | NS231-06 | Australia | Hospital | Paediatric ward (general) | Direct NGS | [**SRX14961725**](https://www.ncbi.nlm.nih.gov/sra/SRX14961725%5Baccn%5D) |
| Sample 4 | NS231-07 | Australia | Hospital | Paediatric ward (general) | Direct NGS | [**SRX14961732**](https://www.ncbi.nlm.nih.gov/sra/SRX14961732%5Baccn%5D) |
| Sample 5 | NS231-08 | Australia | Hospital | Paediatric ward (general) | Direct NGS | [**SRX14961733**](https://www.ncbi.nlm.nih.gov/sra/SRX14961733%5Baccn%5D) |
| Sample 6 | NS231-09 | Australia | Hospital | Paediatric ward (general) | Direct NGS | [**SRX14961734**](https://www.ncbi.nlm.nih.gov/sra/SRX14961734%5Baccn%5D) |
| Sample 7 | NS231-12 | Australia | Hospital | Paediatric ward (general) | Direct NGS | [**SRX14961735**](https://www.ncbi.nlm.nih.gov/sra/SRX14961735%5Baccn%5D) |
| Sample 8 | NS231-14 | Australia | Hospital | Paediatric ward (general) | Direct NGS | [**SRX14961736**](https://www.ncbi.nlm.nih.gov/sra/SRX14961736%5Baccn%5D) |
| Sample 9 | NS231-15 | Australia | Hospital | Paediatric ward (general) | Direct NGS | [**SRX14961737**](https://www.ncbi.nlm.nih.gov/sra/SRX14961737%5Baccn%5D) |
| Sample 10 | NS231-16 | Australia | Hospital | Paediatric ward (general) | Direct NGS | [**SRX14961738**](https://www.ncbi.nlm.nih.gov/sra/SRX14961738%5Baccn%5D) |
| Sample 11 | NS231-19 | Australia | Hospital | Paediatric ward (general) | Direct NGS | [**SRX14961715**](https://www.ncbi.nlm.nih.gov/sra/SRX14961715%5Baccn%5D) |
| Sample 12 | NS250-72 | Australia | Hospital | Paediatric ward (general) | Direct NGS | [**SRX14961716**](https://www.ncbi.nlm.nih.gov/sra/SRX14961716%5Baccn%5D) |
| Sample 13 | NS250-73 | Australia | Hospital | Paediatric ward (general) | Direct NGS | [**SRX14961717**](https://www.ncbi.nlm.nih.gov/sra/SRX14961717%5Baccn%5D) |
| Sample 14 | NS251-34 | Australia | Hospital | Paediatric ward (general) | Direct NGS | [**SRX14961718**](https://www.ncbi.nlm.nih.gov/sra/SRX14961718%5Baccn%5D) |
| Sample 15 | NS300_02 | Australia | Hospital | Paediatric ward (general) | Direct NGS | [**SRX14961721**](https://www.ncbi.nlm.nih.gov/sra/SRX14961721%5Baccn%5D) |
| Sample 16 | NS300_05 | Australia | Hospital | Paediatric ward (general) | Direct NGS | [**SRX14961722**](https://www.ncbi.nlm.nih.gov/sra/SRX14961722%5Baccn%5D) |
| Sample 17 | NS300_06 | Australia | Hospital | Paediatric ward (general) | Direct NGS | [**SRX14961723**](https://www.ncbi.nlm.nih.gov/sra/SRX14961723%5Baccn%5D) |
| Sample 18 | NS300_07 | Australia | Hospital | Paediatric ward (general) | Direct NGS | [**SRX14961724**](https://www.ncbi.nlm.nih.gov/sra/SRX14961724%5Baccn%5D) |
| Sample 19 | NS300_09 | Australia | Hospital | Paediatric ward (general) | Direct NGS | [**SRX14961726**](https://www.ncbi.nlm.nih.gov/sra/SRX14961726%5Baccn%5D) |
| Sample 20 | NS300_10 | Australia | Hospital | Paediatric ward (general) | Direct NGS | [**SRX14961727**](https://www.ncbi.nlm.nih.gov/sra/SRX14961727%5Baccn%5D) |
| Sample 21 | NS300_11 | Australia | Hospital | Paediatric ward (general) | Direct NGS | [**SRX14961728**](https://www.ncbi.nlm.nih.gov/sra/SRX14961728%5Baccn%5D) |
| Sample 22 | NS312-54 | Australia | Hospital | Paediatric ward (general) | Direct NGS | [**SRX14961729**](https://www.ncbi.nlm.nih.gov/sra/SRX14961729%5Baccn%5D) |
| Sample 23 | NS312-56 | Australia | Hospital | Paediatric ward (general) | Direct NGS | [**SRX14961730**](https://www.ncbi.nlm.nih.gov/sra/SRX14961730%5Baccn%5D) |
| Sample 24 | NS313-110 | Australia | Hospital | Paediatric ward (general) | Direct NGS | [**SRX14961731**](https://www.ncbi.nlm.nih.gov/sra/SRX14961731%5Baccn%5D) |
| Sample 25 | R6298_S36 | Australia | Hospital | Paediatric ward (general) | Direct NGS | [**SRX14961719**](https://www.ncbi.nlm.nih.gov/sra/SRX14961719%5Baccn%5D) |
| Sample 26 | R6301_S37 | Australia | Hospital | Paediatric ward (general) | Direct NGS | [**SRX14961720**](https://www.ncbi.nlm.nih.gov/sra/SRX14961720%5Baccn%5D) |
| Sample 27 | ER-01_MBRU2049 | UAE | Hospital | Emergency care unit | Direct NGS | [**SRX11593911**](https://www.ncbi.nlm.nih.gov/sra/SRX11593911%5Baccn%5D) |
| Sample 28 | ER-02_MBRU2049 | UAE | Hospital | Emergency care unit | Direct NGS | [**SRX11593912**](https://www.ncbi.nlm.nih.gov/sra/SRX11593912%5Baccn%5D) |
| Sample 29 | ER-03_MBRU2049 | UAE | Hospital | Emergency care unit | Direct NGS | [**SRX11593923**](https://www.ncbi.nlm.nih.gov/sra/SRX11593923%5Baccn%5D) |
| Sample 30 | ER-04_MBRU2049 | UAE | Hospital | Emergency care unit | Direct NGS | [**SRX11593934**](https://www.ncbi.nlm.nih.gov/sra/SRX11593934%5Baccn%5D) |
| Sample 31 | ER-05_MBRU2049 | UAE | Hospital | Emergency care unit | Direct NGS | [**SRX11593945**](https://www.ncbi.nlm.nih.gov/sra/SRX11593945%5Baccn%5D) |
| Sample 32 | ER-06_MBRU2049 | UAE | Hospital | Emergency care unit | Direct NGS | [**SRX11593953**](https://www.ncbi.nlm.nih.gov/sra/SRX11593953%5Baccn%5D) |
| Sample 33 | ER-07_MBRU2049 | UAE | Hospital | Emergency care unit | Direct NGS | [**SRX11593954**](https://www.ncbi.nlm.nih.gov/sra/SRX11593954%5Baccn%5D) |
| Sample 34 | ER-08_MBRU2049 | UAE | Hospital | Emergency care unit | Direct NGS | [**SRX11593955**](https://www.ncbi.nlm.nih.gov/sra/SRX11593955%5Baccn%5D) |
| Sample 35 | ER-09_MBRU2049 | UAE | Hospital | Emergency care unit | Direct NGS | [**SRX11593956**](https://www.ncbi.nlm.nih.gov/sra/SRX11593956%5Baccn%5D) |
| Sample 36 | ER-10_MBRU2049 | UAE | Hospital | Emergency care unit | Direct NGS | [**SRX11593957**](https://www.ncbi.nlm.nih.gov/sra/SRX11593957%5Baccn%5D) |
| Sample 37 | ER-14A_MBRU2049 | UAE | Hospital | Emergency care unit | Direct NGS | [**SRX11593913**](https://www.ncbi.nlm.nih.gov/sra/SRX11593913%5Baccn%5D) |
| Sample 38 | ER-15_MBRU2049 | UAE | Hospital | Emergency care unit | Direct NGS | [**SRX11593915**](https://www.ncbi.nlm.nih.gov/sra/SRX11593915%5Baccn%5D) |
| Sample 39 | ER-16_MBRU2049 | UAE | Hospital | Emergency care unit | Direct NGS | [**SRX11593944**](https://www.ncbi.nlm.nih.gov/sra/SRX11593944%5Baccn%5D) |
| Sample 40 | ER-18_MBRU2049 | UAE | Hospital | Emergency care unit | Direct NGS | [**SRX11593916**](https://www.ncbi.nlm.nih.gov/sra/SRX11593916%5Baccn%5D) |
| Sample 41 | ER-19_MBRU2049 | UAE | Hospital | Emergency care unit | Direct NGS | [**SRX11593917**](https://www.ncbi.nlm.nih.gov/sra/SRX11593917%5Baccn%5D) |
| Sample 42 | ER-20_MBRU2049 | UAE | Hospital | Emergency care unit | Direct NGS | [**SRX11593918**](https://www.ncbi.nlm.nih.gov/sra/SRX11593918%5Baccn%5D) |
| Sample 43 | ER-23_MBRU2049 | UAE | Hospital | Emergency care unit | Direct NGS | [**SRX11593919**](https://www.ncbi.nlm.nih.gov/sra/SRX11593919%5Baccn%5D) |
| Sample 44 | ER-24_MBRU2049 | UAE | Hospital | Emergency care unit | Direct NGS | [**SRX11593920**](https://www.ncbi.nlm.nih.gov/sra/SRX11593920%5Baccn%5D) |
| Sample 45 | ER-25_MBRU2049 | UAE | Hospital | Emergency care unit | Direct NGS | [**SRX11593921**](https://www.ncbi.nlm.nih.gov/sra/SRX11593921%5Baccn%5D) |
| Sample 46 | ER-26_MBRU2049 | UAE | Hospital | Emergency care unit | Direct NGS | [**SRX11593922**](https://www.ncbi.nlm.nih.gov/sra/SRX11593922%5Baccn%5D) |
| Sample 47 | ER-27A_MBRU2049 | UAE | Hospital | Emergency care unit | Direct NGS | [**SRX11593924**](https://www.ncbi.nlm.nih.gov/sra/SRX11593924%5Baccn%5D) |
| Sample 48 | ER-28_MBRU2049 | UAE | Hospital | Emergency care unit | Direct NGS | [**SRX11593926**](https://www.ncbi.nlm.nih.gov/sra/SRX11593926%5Baccn%5D) |
| Sample 49 | ER-29_MBRU2049 | UAE | Hospital | Emergency care unit | Direct NGS | [**SRX11593927**](https://www.ncbi.nlm.nih.gov/sra/SRX11593927%5Baccn%5D) |
| Sample 50 | ER-30_MBRU2049 | UAE | Hospital | Emergency care unit | Direct NGS | [**SRX11593928**](https://www.ncbi.nlm.nih.gov/sra/SRX11593928%5Baccn%5D) |
| Sample 51 | ER-34_MBRU2049 | UAE | Hospital | Emergency care unit | Direct NGS | [**SRX11593946**](https://www.ncbi.nlm.nih.gov/sra/SRX11593946%5Baccn%5D) |
| Sample 52 | ER-36_MBRU2049 | UAE | Hospital | Emergency care unit | Direct NGS | [**SRX11593947**](https://www.ncbi.nlm.nih.gov/sra/SRX11593947%5Baccn%5D) |
| Sample 53 | ER-39_MBRU2049 | UAE | Hospital | Emergency care unit | Direct NGS | [**SRX11593948**](https://www.ncbi.nlm.nih.gov/sra/SRX11593948%5Baccn%5D) |
| Sample 54 | ER-40_MBRU2049 | UAE | Hospital | Emergency care unit | Direct NGS | [**SRX11593929**](https://www.ncbi.nlm.nih.gov/sra/SRX11593929%5Baccn%5D) |
| Sample 55 | ER-41A_MBRU2049 | UAE | Hospital | Emergency care unit | Direct NGS | [**SRX11593930**](https://www.ncbi.nlm.nih.gov/sra/SRX11593930%5Baccn%5D) |
| Sample 56 | ER-43_MBRU2049 | UAE | Hospital | Emergency care unit | Direct NGS | [**SRX11593932**](https://www.ncbi.nlm.nih.gov/sra/SRX11593932%5Baccn%5D) |
| Sample 57 | ER-44_MBRU2049 | UAE | Hospital | Emergency care unit | Direct NGS | [**SRX11593933**](https://www.ncbi.nlm.nih.gov/sra/SRX11593933%5Baccn%5D) |
| Sample 58 | ER-45_MBRU2049 | UAE | Hospital | Emergency care unit | Direct NGS | [**SRX11593935**](https://www.ncbi.nlm.nih.gov/sra/SRX11593935%5Baccn%5D) |
| Sample 59 | ER-46_MBRU2049 | UAE | Hospital | Emergency care unit | Direct NGS | [**SRX11593936**](https://www.ncbi.nlm.nih.gov/sra/SRX11593936%5Baccn%5D) |
| Sample 60 | ER-48_MBRU2049 | UAE | Hospital | Emergency care unit | Direct NGS | [**SRX11593937**](https://www.ncbi.nlm.nih.gov/sra/SRX11593937%5Baccn%5D) |
| Sample 61 | ER-50_MBRU2049 | UAE | Hospital | Emergency care unit | Direct NGS | [**SRX11593938**](https://www.ncbi.nlm.nih.gov/sra/SRX11593938%5Baccn%5D) |
| Sample 62 | ER-54A_MBRU2049 | UAE | Hospital | Emergency care unit | Direct NGS | [**SRX11593939**](https://www.ncbi.nlm.nih.gov/sra/SRX11593939%5Baccn%5D) |
| Sample 63 | ER-55_MBRU2049 | UAE | Hospital | Emergency care unit | Direct NGS | [**SRX11593941**](https://www.ncbi.nlm.nih.gov/sra/SRX11593941%5Baccn%5D) |
| Sample 64 | ER-58_MBRU2049 | UAE | Hospital | Emergency care unit | Direct NGS | [**SRX11593942**](https://www.ncbi.nlm.nih.gov/sra/SRX11593942%5Baccn%5D) |
| Sample 65 | ER-59_MBRU2049 | UAE | Hospital | Emergency care unit | Direct NGS | [**SRX11593943**](https://www.ncbi.nlm.nih.gov/sra/SRX11593943%5Baccn%5D) |
| Sample 66 | NS309-13 | Australia | Hospital | PICU | Indirect NGS (Viable) | [**SRX33059931**](https://www.ncbi.nlm.nih.gov/sra/SRX33059931%5Baccn%5D) |
| Sample 67 | NS309-14 | Australia | Hospital | PICU | Indirect NGS (Viable) | [**SRX33059932**](https://www.ncbi.nlm.nih.gov/sra/SRX33059932%5Baccn%5D) |
| Sample 68 | NS309-15 | Australia | Hospital | PICU | Indirect NGS (Viable) | [**SRX33059943**](https://www.ncbi.nlm.nih.gov/sra/SRX33059943%5Baccn%5D) |
| Sample 69 | NS309-16 | Australia | Hospital | PICU | Indirect NGS (Viable) | [**SRX33059954**](https://www.ncbi.nlm.nih.gov/sra/SRX33059954%5Baccn%5D) |
| Sample 70 | NS309-17 | Australia | Hospital | PICU | Indirect NGS (Viable) | [**SRX33059955**](https://www.ncbi.nlm.nih.gov/sra/SRX33059955%5Baccn%5D) |
| Sample 71 | NS309-18 | Australia | Hospital | NICU | Indirect NGS (Viable) | [**SRX33059956**](https://www.ncbi.nlm.nih.gov/sra/SRX33059956%5Baccn%5D) |
| Sample 72 | NS309-19 | Australia | Hospital | NICU | Indirect NGS (Viable) | [**SRX33059957**](https://www.ncbi.nlm.nih.gov/sra/SRX33059957%5Baccn%5D) |
| Sample 73 | NS309-20 | Australia | Hospital | NICU | Indirect NGS (Viable) | [**SRX33059958**](https://www.ncbi.nlm.nih.gov/sra/SRX33059958%5Baccn%5D) |
| Sample 74 | NS309-21 | Australia | Hospital | NICU | Indirect NGS (Viable) | [**SRX33059959**](https://www.ncbi.nlm.nih.gov/sra/SRX33059959%5Baccn%5D) |
| Sample 75 | NS309-22 | Australia | Hospital | NICU | Indirect NGS (Viable) | [**SRX33059960**](https://www.ncbi.nlm.nih.gov/sra/SRX33059960%5Baccn%5D) |
| Sample 76 | NS309-23 | Australia | Hospital | PED | Indirect NGS (Viable) | [**SRX33059933**](https://www.ncbi.nlm.nih.gov/sra/SRX33059933%5Baccn%5D) |
| Sample 77 | NS309-24 | Australia | Hospital | PED | Indirect NGS (Viable) | [**SRX33059934**](https://www.ncbi.nlm.nih.gov/sra/SRX33059934%5Baccn%5D) |
| Sample 78 | NS309-25 | Australia | Hospital | PED | Indirect NGS (Viable) | [**SRX33059935**](https://www.ncbi.nlm.nih.gov/sra/SRX33059935%5Baccn%5D) |
| Sample 79 | NS309-26 | Australia | Hospital | PED | Indirect NGS (Viable) | [**SRX33059936**](https://www.ncbi.nlm.nih.gov/sra/SRX33059936%5Baccn%5D) |
| Sample 80 | NS309-27 | Australia | Hospital | PED | Indirect NGS (Viable) | [**SRX33059937**](https://www.ncbi.nlm.nih.gov/sra/SRX33059937%5Baccn%5D) |
| Sample 81 | R8911_S8 | Australia | Hospital | PED | Indirect NGS (Viable) | [**SRX33059938**](https://www.ncbi.nlm.nih.gov/sra/SRX33059938%5Baccn%5D) |
| Sample 82 | R8912_S9 | Australia | Hospital | PED | Indirect NGS (Viable) | [**SRX33059939**](https://www.ncbi.nlm.nih.gov/sra/SRX33059939%5Baccn%5D) |
| Sample 83 | R8913_S10 | Australia | Hospital | PED | Indirect NGS (Viable) | [**SRX33059940**](https://www.ncbi.nlm.nih.gov/sra/SRX33059940%5Baccn%5D) |
| Sample 84 | R8914_S11 | Australia | Hospital | PED | Indirect NGS (Viable) | [**SRX33059941**](https://www.ncbi.nlm.nih.gov/sra/SRX33059941%5Baccn%5D) |
| Sample 85 | R8915_S12 | Australia | Hospital | PED | Indirect NGS (Viable) | [**SRX33059942**](https://www.ncbi.nlm.nih.gov/sra/SRX33059942%5Baccn%5D) |
| Sample 86 | R8916_S13 | Australia | Hospital | PED | Indirect NGS (Viable) | [**SRX33059944**](https://www.ncbi.nlm.nih.gov/sra/SRX33059944%5Baccn%5D) |
| Sample 87 | R8917_S14 | Australia | Hospital | PED | Indirect NGS (Viable) | [**SRX33059945**](https://www.ncbi.nlm.nih.gov/sra/SRX33059945%5Baccn%5D) |
| Sample 88 | R8918_S15 | Australia | Hospital | PED | Indirect NGS (Viable) | [**SRX33059946**](https://www.ncbi.nlm.nih.gov/sra/SRX33059946%5Baccn%5D) |
| Sample 89 | R8919_S16 | Australia | Hospital | PED | Indirect NGS (Viable) | [**SRX33059947**](https://www.ncbi.nlm.nih.gov/sra/SRX33059947%5Baccn%5D) |
| Sample 90 | R8920_S17 | Australia | Hospital | PED | Indirect NGS (Viable) | [**SRX33059948**](https://www.ncbi.nlm.nih.gov/sra/SRX33059948%5Baccn%5D) |
| Sample 91 | R8921_S18 | Australia | Hospital | PED | Indirect NGS (Viable) | [**SRX33059949**](https://www.ncbi.nlm.nih.gov/sra/SRX33059949%5Baccn%5D) |
| Sample 92 | R8922_S19 | Australia | Hospital | PED | Indirect NGS (Viable) | [**SRX33059950**](https://www.ncbi.nlm.nih.gov/sra/SRX33059950%5Baccn%5D) |
| Sample 93 | R8923_S20 | Australia | Hospital | PED | Indirect NGS (Viable) | [**SRX33059951**](https://www.ncbi.nlm.nih.gov/sra/SRX33059951%5Baccn%5D) |
| Sample 94 | R8924_S21 | Australia | Hospital | PED | Indirect NGS (Viable) | [**SRX33059952**](https://www.ncbi.nlm.nih.gov/sra/SRX33059952%5Baccn%5D) |
| Sample 95 | R8925_S22 | Australia | Hospital | PED | Indirect NGS (Viable) | [**SRX33059953**](https://www.ncbi.nlm.nih.gov/sra/SRX33059953%5Baccn%5D) |
